# Influence of playing standard and physical fitness on activity profiles and post-match fatigue during intensified junior rugby league competition

**DOI:** 10.1186/s40798-015-0015-y

**Published:** 2015-04-03

**Authors:** Rich D Johnston, Tim J Gabbett, David G Jenkins

**Affiliations:** 1 School of Exercise Science, Australian Catholic University, Brisbane, Queensland 4014 Australia; 2 School of Human Movement Studies, University of Queensland, Brisbane, Queensland 4072 Australia

**Keywords:** Neuromuscular fatigue, Muscle damage, Contact sports, GPS, Physical demands

## Abstract

**Background:**

The aim of this study was to determine whether the fatigue responses to the same intensified rugby league competition differed depending on playing standard and physical fitness.

**Methods:**

Players from a high-standard (*n* = 15) and a low-standard (*n* = 16) junior rugby league team had lower body neuromuscular fatigue, perceptual wellbeing, and blood creatine kinase (CK) assessed over an intensified competition. Global positioning system units measured match activity profiles and rating of perceived exertion-assessed internal loads. Players were divided into high- and low-fitness groups across the two standards based on Yo-Yo intermittent recovery test performance.

**Results:**

Playing intensity increased with playing standard and fitness levels (high-standard = 92 ± 6 m·min^−1^ vs. 88 ± 6 m·min^−1^; low-standard = 88 ± 2 m·min^−1^ vs. 83 ± 6 m·min^−1^). Despite greater internal and external loads, high-fitness players showed smaller reductions in lower body power (high-standard effect size [ES] = −0.74; low-standard ES = −0.41). High-standard players had smaller increases in blood CK (77% ± 94% vs. 113% ± 81%; ES = −0.41), primarily due to very small increases in the high-fitness group (50% ± 45%).

**Conclusions:**

Increased fitness leads to greater internal and external workloads during intensified competition, smaller increases in blood CK, and less neuromuscular fatigue. Maximising player fitness should be a primary goal of coaches in order to increase match workloads and reduce post-match fatigue during intensified competition.

**Key Points:**

- Increased physical fitness results in greater relative and absolute match workloads.
- Increased physical fitness results in less fatigue and muscle damage during an intensified competition.
- Coaching staff should aim to maximise physical fitness in order to optimise match performance and reduce player fatigue.

## Background

Rugby league is a physically demanding sport that involves periods of high-intensity activity (e.g. high-speed running, sprinting, physical contact) interspersed with periods of low-intensity activity (e.g. standing, walking, jogging) [1]. During competition, players typically cover relative distances of 90 to 100 m·min^−1^ [1-3], which increases with playing standard [4]. In addition to these running demands, players frequently engage in physical contact (i.e. tackles, hit-ups, and wrestles) during attack and defence [2]. Gabbett et al. [5] reported that players performed 24 to 47 contact efforts during a game at an average frequency of 0.38 to 1.09 contacts·min^−1^, although players can perform up to 1.9 ± 0.7 contacts·min^−1^ depending on playing position, phase of play, and field position [6].

Given the intense physical demands of rugby league, match play rugby results in increased markers of muscle damage, as well as neuromuscular and perceptual fatigue [7-9]. Whilst this fatigue is generally transient in nature, typically persisting for 24 to 48 h after competition, muscle damage can last for 5 days [8]. As such, during tournaments or periods of congested fixtures when players may be required to play multiple games within a week, insufficient recovery may occur [10]. Indeed, during rugby league [10,11], basketball [12], and soccer tournaments [13], fatigue accumulates, which compromises high-intensity match activities during the latter stages of the competition. Johnston et al. [11] showed that the relative distance covered at high speeds was reduced by 50% and 60% in the final two games of an intensified rugby league tournament. Whilst studies from basketball and soccer suggest that recovery strategies, and in particular cold water immersion, may be useful to minimise fatigue-mediated reductions in performance during intensified competitions [12-14], physical qualities such as high-intensity running ability and lower body strength also appear to play an important role in minimising post-match fatigue in rugby league players [9]. Although physical qualities may attenuate post-match fatigue following regular games [9], it is unclear whether they could help minimise fatigue that may occur during intensified rugby league competition [10,11].

It is well documented that the intensity of match play increases with competitive standard [4,15,16]. Indeed, Gabbett reported that, during a junior rugby league tournament, first division players covered greater metres per minute at both low and high speeds and engaged in a higher frequency of collisions and repeated high-intensity effort (RHIE) bouts than third division players [15]. As such, it would seem logical that increased playing intensity would lead to greater fatigue; however, this may be offset by enhanced physical qualities [9]. As playing standard increases in junior players, so too do physical qualities [17,18], which appear central to minimising post-match fatigue [9]. With this in mind, the aims of this study were to investigate whether there was a difference between (1) fatigue responses based on playing level, (2) fatigue responses based on physical fitness, and (3) match activity profiles based on playing standard and physical fitness during the same intensified rugby league competition. It was hypothesised that players competing in the first division (high-standard) would have smaller increases in fatigue despite increased match intensity than players competing in the third division (low-standard) of the competition. In addition, we hypothesised that players from both high- and low-standard teams with well-developed physical qualities would experience less fatigue and greater workloads over the course of the competition.

## Methods

### Experimental design

In order to test our hypotheses, a between groups, repeated measures experimental design was used. Players from two junior teams (one high- and one low-standard) were tracked for markers of fatigue (neuromuscular and perceptual wellbeing) and muscle damage (blood creatine kinase [CK]) during an intensified rugby league competition. In addition, global positioning system (GPS) microtechnology provided information on the activity profiles of players during matches. To assess the impact of physical fitness on match activities and post-match fatigue, players were also divided into high- and low-fitness groups based on their Yo-Yo intermittent recovery rest (IRT) level 1 performance.

### Participants

Thirty-one junior rugby league players (age 16.5 ± 0.5 years; body mass 79.6 ± 11.6 kg) competing for two separate schools in the 2014 Confraternity Shield tournament volunteered to participate in the study. One team was competing in the first division of the competition and represented the high-standard team (*entire team*, *n* = 15; age 16.6 ± 0.5 years; body mass 78.5 ± 9.9 kg; *high-fitness group*, *n* = 8; age 16.6 ± 0.6 years; body mass 78.2 ± 8.8 kg; *low-fitness group*, *n* = 7; age 16.5 ± 0.6 years; body mass 78.9 ± 11.2 kg), the second team was competing in the third division, representing the low-standard team (*entire team*, *n* = 16; age 16.5 ± 0.6 years; body mass 79.6 ± 13.4 kg; *high-fitness group*, *n* = 8; age 16.3 ± 0.4 years; body mass 77.2 ± 10.8 kg; *low-fitness group*, *n* = 8; age 16.7 ± 0.6 years; body mass 84.8 ± 16.6 kg). The tournament took place in July, 3 months into the competitive season. All players were free from injury at the time of testing and were asked to maintain their normal diet throughout the competition; water was available *ad libitum* throughout. Before the study, players attended a familiarisation session and received an information sheet outlining experimental procedures, and the associated risks and benefits of participation. In accordance with the Code of Ethics of the World Medical Association (Declaration of Helsinki), players received an information sheet outlining experimental procedures; written informed consent was obtained from each player and their legal guardian. The study was approved by the Australian Catholic University ethical review board for human research.

### Protocol

**Figure 1:**
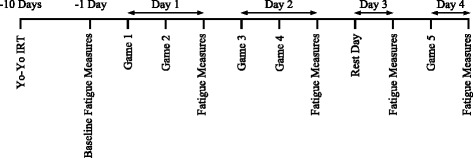
**A schematic overview of the data collection protocol.** Yo-Yo IRT = Yo-Yo intermittent recovery test (level 1); fatigue measures were conducted within 1 h after the final game of each day and included a countermovement jump, blood creatine kinase, and perceptual wellbeing.

Ten days prior to the tournament, the Yo-Yo IRT level 1 was used to assess high-intensity intermittent running ability [19]. The test was performed at 15:00 hrs on a grassed playing surface at the start of a training session; players wore studded boots and training kit to complete the test. The test requires players to complete a 20-m shuttle at progressively faster speeds whilst keeping in time with an audio signal. Between each shuttle, there is a 10-s period of active recovery involving a jog/walk around a cone placed 5 m from the start/finish line. When players failed to keep in time with the audio signals on two consecutive occasions, they were deemed to have failed the test; the last level successfully completed and the corresponding metres covered were used as the final score for the test. The players were asked to maintain their normal diet and refrain from physical activity during the 24 h prior to the test. Some of the players were unfamiliar with the test so the first two levels of the test were incorporated into the warm-up to familiarise players with the test protocol. The typical error of measurement (TE) for this test is 4.9% [19]. A schematic overview of the protocol can be seen in Figure 1.

The first 4 games of the tournament were 40 min (2 × 20 min halves), with the final game being 50 min (2 × 25 min halves) in duration. Two games were played on both days 1 and 2, no games on day 3, and one game on day 4, totalling five games (210 min) over a 4-day period. Baseline measurements of neuromuscular fatigue, muscle damage, and perceived wellbeing were assessed approximately 12 h prior to game 1. Subsequent fatigue and muscle damage measures were taken within 1 h of the final match of each day. On day 3, when no games were played, fatigue measures were assessed at the same time as they were on other days (approximately 14:00 to 16:00 hrs). The average temperature, rainfall, and humidity over the competition period were 20.6°C ± 2.2°C, 0.00 ± 0.00 mm, and 51.0% ± 15.3%, respectively.

### Neuromuscular fatigue

Lower body neuromuscular fatigue was assessed using peak power from a single countermovement jump (CMJ) performed on a force platform (Kistler 9290AD Force Platform, Kistler, Amherst, NY, USA) interfaced with a laptop (Acer Aspire 2930, Acer, Telford, UK) running manufacturer-designed software (QuattroJump, Kistler, Amherst, NY, USA) [10]. Players kept their hands on their hips throughout the jump; no instruction was given on the depth of the countermovement, but players were asked to jump as high as possible on the experimenter’s signal in line with previous methodology [10]. The TE from our laboratory for CMJ peak power is 3.5%. Peak power was used to assess neuromuscular fatigue as it offers good reliability and ensures consistency with previous rugby league literature [8-10,20-22].

### Creatine kinase

Whole-blood CK activity was used as a marker of muscle damage. A 30-μl sample of blood was taken from a fingertip and immediately analysed using a colorimetric assay procedure (Reflotron, Boehringer Mannheim, Germany). Before each testing session, the instrument was calibrated in accordance with the manufacturer recommendations. The ‘normal’ reference range for CK activity, as provided by the manufacturer using this method, is 24 to 195 IU·l^−1^ [7,10,23]. The TE for assessing CK was 3.3%.

### Perceptual wellbeing

Each day, perceived wellbeing was assessed by the experimenter asking players to rate feelings of fatigue, lower and upper body muscle soreness, sleep quality, and mood and stress on 0 to 5 Likert scales, with the individual scores summated to give an overall wellbeing score using methods outlined previously [10]. The test re-test reliability for the perceptual wellbeing questionnaire was determined by having 12 rugby league players complete the questionnaire on two separate occasions, 7 days apart following 36 h of no physical activity; the TE for perceptual wellbeing was 3.0%. Additionally, 30 min after each game, rating of perceived exertion (RPE) was recorded using a modified RPE scale (CR-10) to rate how hard players perceived each game. The RPE score was then multiplied by the number of minutes played to determine session RPE as a measure of internal load [24]. This method of assessing internal loads has shown to have appropriate levels of validity and reliability (TE = 4.0%) in rugby league players [25].

### Match activity profiles

Activity profiles during competition were assessed by GPS analysis. Prior to the warm-up before each game, players were fitted with the GPS vest and unit; the unit was switched on and inserted into a padded compartment at the rear of the vest, positioned between the shoulder blades. The GPS units sampled at 10 Hz (Team S4, Catapult Sports, Victoria, Australia) and included a triaxial accelerometer and gyroscope sampling at 100 Hz to provide information on collisions. Data were downloaded to a laptop (Acer Aspire 2930, Acer, Telford, UK) and analysed using software provided by the manufacturer (Sprint, Version 5.1.1, Catapult Sports, Victoria, Australia). Non-playing minutes were omitted from the analysis. Data were categorised into low- (0 to 3.5 m·s^−1^), moderate- (3.6 to 5.0 m·s^−1^) and high-speed (≥5.1 m·s^−1^) movement bands [26]; the total number of collisions was recorded as described previously [1]. In addition, the average speed of each game (m·min^−1^) was calculated in relation to the final speed reached by players on the Yo-Yo IRT (m·min^−1^) in order to express the speed of the match in relation to the physical capacity of each player. RHIE bouts were classified as three or more maximal acceleration (≥2.78 m · s^−2^), high-speed, or impact efforts with less than 21 s between each effort [1]. These units are reliable for quantifying movements commonplace in rugby league [27-29].

### Statistical analysis

In order to determine the influence of physical fitness on match activities and post-match fatigue, players from both the high- and low-standard team were matched for position (forwards and backs) before being divided into low- and high-fitness groups, using a median split, based on Yo-Yo IRT performance. This ensured that there was an even number of forwards and backs in both fitness groups so that the fatigue responses were not influenced by the different match activities performed by each positional group. This provided us with four experimental groups based on Yo-Yo IRT performance: high-standard/high-fitness, high-standard/low-fitness, low-standard/high-fitness, and low-standard/low-fitness.

Differences in fatigue, muscle damage, and activity profiles between the high- and low-standard and high- and low-fitness playing groups and changes over time were determined using traditional null hypothesis testing and magnitude-based inferences. To compare differences in Yo-Yo IRT performance and match activity profiles between high- and low-standard playing groups and fitness levels, a two-way group (high- vs. low-standard) × fitness (high-standard/high-fitness vs. high-standard/low-fitness vs. low-standard/high-fitness vs. low-standard/low-fitness) ANOVA was used. A three-way group (high- vs. low-standard) × time (baseline vs. day 1 vs. 2 vs. 3 vs. 4) × fitness (high-standard/high-fitness vs. high-standard/low-fitness vs. low-standard/high-fitness vs. low-standard/low-fitness) repeated measures ANOVA (SPSS 22.0, SPSS Inc, Chicago, IL, USA) was used to determine changes in neuromuscular fatigue, blood CK, and activity profiles between playing standards and fitness levels. If significant main effects were found, Bonferroni *post hoc* analyses were performed to locate the differences. Changes and differences in perceptual wellbeing and RPE were analysed using a Kruskal-Wallis test. Based on the real-world relevance of the results, magnitude-based inferences were used to assess the meaningfulness of any differences. Firstly, the likelihood that changes in the dependent variables were greater than the smallest worthwhile change was calculated as a small effect size of 0.20 × between subject standard deviation. Based on 90% confidence intervals, the thresholds used for assigning qualitative terms to chances were as follows: <1% almost certainly not; <5% very unlikely; <25% unlikely; <50% possibly not; >50% possibly; >75% likely; >95% very likely; >99% almost certain [30]. The magnitude of difference was considered practically meaningful when the likelihood was ≥75%. Secondly, magnitudes of change in the dependent variables were assessed using Cohen’s effect size (ES) statistic [31]. ES of 0.20 to 0.60, 0.61 to 1.19, and ≥1.20 were considered small, moderate, and large, respectively [32]. Data are reported as means ± standard deviation (SD); the significance level was set at *p* < 0.05.

## Results

### Match activity profiles

#### Playing standard and activity profiles

There were a number of differences in match activity profiles between the high- and low-standard playing groups (Table 1). The greatest differences were seen in absolute workloads across the five games, primarily due to the high-standard players completing more playing minutes. Despite this, there was still greater relative distance (*p* = 0.05) covered at both high (*p* = 0.224) and moderate speeds (*p* = 0.02) in the high-standard group, more frequent collisions (*p* = 0.019) and RHIE bouts (*p* = 0.004), and greater internal loads highlighted by session RPE (*p* = 0.001). High-standard players covered significantly greater distances on the Yo-Yo IRT (*p* = 0.001). When match speed (m·min^−1^) was expressed relative to maximal Yo-Yo IRT speed, there was no difference in match intensity (*p* = 0.75).

**Table 1:** **Physical qualities and average match activity profiles across the tournament between the high- and low-standard team**

	**High-standard**	**Low-standard**	**ES**	**Likelihood**
Playing time (min)	36 ± 6	30 ± 8	0.85	96%, very likely
Distance covered (m)	3,327 ± 588*	2,516 ± 720	1.24	100%, almost certain
Relative distance (m·min^−1^)	90 ± 7*	85 ± 7	0.71	92%, likely
Low-speed activity (m)	2,525 ± 444	2,074 ± 576	0.88	96%, very likely
Low-speed activity (m·min^−1^)	71 ± 5	69 ± 6	0.19	56%, possibly
Moderate-speed running (m)	434 ± 115*	274 ± 94	1.52	100%, almost certain
Moderate-speed running (m·min^−1^)	13 ± 3*	9 ± 2	1.58	100%, almost certain
High-speed running (m)	174 ± 51*	116 ± 58	1.07	99%, very likely
High- speed running (m·min^−1^)	4.3 ± 1.1	3.8 ± 1.0	0.52	81%, likely
Match speed vs. Yo-Yo speed (%)	34 ± 3	34 ± 3	0.05	25%, unlikely
*Collisions*				
Total (no.)	15 ± 7*	9 ± 3	1.30	100%, almost certain
Total (no./min)	0.4 ± 0.2*	0.3 ± 0.1	0.72	76%, likely
*Repeated high-intensity efforts*				
Bouts (no.)	3.2 ± 1.8*	1.3 ± 0.6	1.43	100%, almost certain
Bout frequency (no./min)	1 every 17 min*	1 every 23 min	0.62	85%, likely
*Internal Loads*				
RPE (AU)	5 ± 2	3 ± 1	1.06	98%, very likely
Session RPE (AU)	180 ± 39*	109 ± 37	1.90	100%, almost certain
*Physical Qualities*				
Yo-Yo IRT (m)	1,420 ± 337*	922 ± 227	1.73	100%, almost certain

Low-speed activity = 0 to 3.5 m·s^−1^; moderate-speed running = 3.6 to 5.0 m·s^−1^; high-speed running = ≥5.1 m·s^−1^. RPE = rating of perceived exertion; session RPE = playing time × RPE; Yo-Yo IRT = Yo-Yo intermittent recovery test level 1. ES = effect size, 0.20 to 0.60, 0.61 to 1.19, and >1.20 were considered small, moderate, and large, respectively. Likelihoods ≥75% are classified as practically meaningful. *Denotes a statistically significant difference (*p* < 0.05) between playing standards.

#### Physical fitness and activity profiles

When players from both the high- and low-standard teams were divided into low- and high-fitness groups, there were further differences in physical match performance variables (Table 2). High-fitness players covered more metres per minute of match play across both playing standards and had greater internal loads. High-standard/high-fitness players covered greater distances than all other groups primarily through increased distance covered at moderate speeds (*p* < 0.05). In addition, they engaged in more collisions per minute and RHIE bouts (*p* < 0.05). Whilst there were a number of differences between the high-standard fitness groups, the differences between high- and low-fitness groups were not as great in the low-standard playing group with only moderate differences in relative distance, primarily achieved by greater relative distances covered at low and moderate speeds, and higher internal loads in the high-fitness group. When match speed (m·min^−1^) was expressed relative to maximal Yo-Yo IRT speed, the high-fitness groups maintained a greater relative intensity across the tournament (high-standard: ES = 0.61; likelihood = 77%, likely; low-standard: ES = 0.22; likelihood = 52%, possibly). The high-standard/high-fitness playing group covered more metres on the Yo-Yo IRT than the low-standard/high-fitness group (ES = 3.88; likelihood = 100%, almost certain). Both high-standard/high-fitness and high-standard/low-fitness players had greater session RPE loads across the competition compared with both low-standard groups (*p* = 0.001).

**Table 2:** **Physical qualities and average match activity profiles across the tournament between high- and low-fitness groups**

	**High-standard**			**Low-standard**		
	**High-fitness**	**Low-fitness**	**ES**	**High-fitness**	**Low-fitness**	**ES**
Playing time (min)	38 ± 3	33 ± 4	1.41	31 ± 10	30 ± 7	0.16
Distance covered (m)	3,541 ± 278	2,943 ± 735	1.08	2,716 ± 928	2,455 ± 478*	0.35
Relative distance (m·min^−1^)	92 ± 6	88 ± 6	0.73	88 ± 2	83 ± 6*	1.04
Low-speed activity (m)	2,642 ± 318	2,275 ± 537	0.83	2,218 ± 752	2,007 ± 407	0.35
Low-speed activity (m·min^−1^)	70 ± 6	70 ± 3	0.04	72 ± 3	68 ± 6	0.69
Moderate-speed running (m)	503 ± 106	349 ± 104*	1.46	302 ± 88*	258 ± 49*	0.62
Moderate-speed running (m·min^−1^)	13 ± 3	11 ± 2	0.99	10 ± 2*	9 ± 2*	0.36
High-speed running (m)	183 ± 41	149 ± 53	0.74	119 ± 56	117 ± 67	0.03
High-speed running (m·min^−1^)	4.5 ± 1.1	4.0 ± 1.0	0.45	3.8 ± 0.8	3.8 ± 1.3	0.05
Match speed vs. Yo-Yo speed (%)	35 ± 3	33 ± 2	0.61	35 ± 2	34 ± 3	0.22
*Collisions*						
Total (no.)	19 ± 6	13 ± 7	1.24	10 ± 3*	9 ± 3*	0.35
Total (no./min)	0.5 ± 0.2	0.4 ± 0.1	0.85	0.3 ± 0.1*	0.3 ± 0.1*	0.19
*Repeated high-intensity efforts*						
Bouts (no.)	4 ± 2	3 ± 1*	0.79	1 ± 1*	1 ± 1*	−0.23
Bout frequency (no./min)	1 every 14 min	1 every 16 min	0.26	1 every 24 min	1 every 22 min	−0.27
*Internal Loads*						
RPE (AU)	5 ± 2	5 ± 1	−0.02	3 ± 1	3 ± 1	0.24
Session RPE (AU)	196 ± 31	174 ± 37	0.65	116 ± 22*†	100 ± 27*†	0.64
*Physical Qualities*						
Yo-Yo IRT (m)	1,700 ± 119	1,233 ± 304*	2.02	1,089 ± 188*†	785 ± 155*†	1.76

Low-speed activity = 0 to 3.5 m·s^−1^; moderate-speed running = 3.6 to 5.0 m·s^−1^; high-speed running = ≥5.1 m·s^−1^. RPE = rating of perceived exertion; session RPE = playing time × RPE; Yo-Yo IRT = Yo-Yo intermittent recovery test level 1. ES = effect size, 0.20 to 0.60, 0.61 to 1.19, and >1.20 were considered small, moderate, and large respectively. *Denotes a statistically significant difference (*p* < 0.05) from the high-standard/high-fitness group; †Denotes a statistically significant difference from the high-standard/low-fitness group.

### Fatigue

#### Playing standard and fatigue

**Figure 2:**
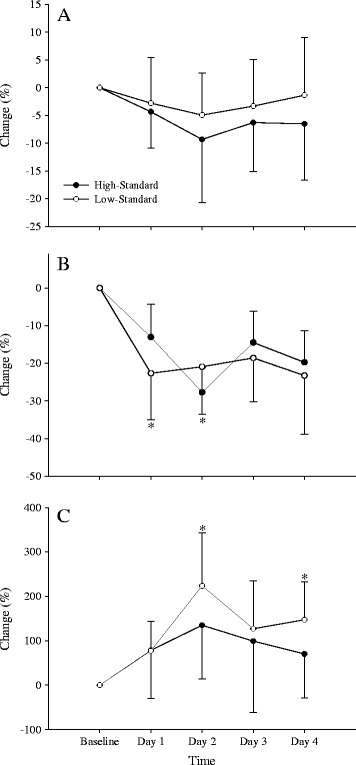
**Changes in CMJ peak power, perceptual wellbeing, and blood creatine kinase between high/low-standard playing groups.** Changes in **(A)** countermovement jump peak power, **(B)** perceptual wellbeing, and **(C)** blood creatine kinase between the high- and low-standard playing groups over the course of the intensified competition. *Denotes a moderate effect size difference (0.60 to 1.20) between the high- and low-standard playing groups.

There was little difference in the fatigue responses between the high- and low-standard players across the course of the competition (Figure 2). There were significant reductions in CMJ power over the competition (*p* = 0.004) which peaked on day 2 but only small differences (ES = 0.21 to 0.50; *p* = 0.581) between high- and low-standard players (Figure 2A). Blood CK increased over the competition (*p* = 0.01), peaking on day 2 in both playing groups (Figure 2C). There was a greater overall increase across the competition in the low-standard players (76% ± 94% vs. 113% ± 81%; ES = 0.83, likelihood = 76%, likely; *p* = 0.078), as well as moderately greater increases on day 2 (ES = 0.74, likelihood = 89%, likely; *p* = 0.012) and day 4 (ES = 0.84, likelihood = 99%, very likely; *p* = 0.015). There were significant reductions in perceived wellbeing over the competition (Figure 2B [*p* = 0.001]), which was similar between the high- and low-standard players (−14% ± 17% vs. −17% ± 9%; ES = 0.40, likelihood = 71%, possibly), with no significant difference between playing standards on any day of the competition.

#### Physical fitness and fatigue

**Figure 3:**
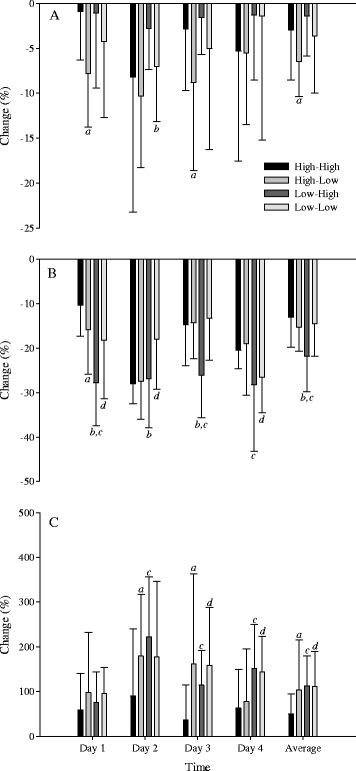
**Fatigue changes with players from high- and low-standard playing groups, divided into low- and high-fitness groups.** Changes in **(A)** countermovement jump peak power, **(B)** perceptual wellbeing, and **(C)** blood creatine kinase with players from the high- and low-standard playing groups, divided into low- and high-fitness groups over the course of the intensified competition. High-High = high-playing standard/high fitness; High-Low = high-playing standard/low fitness; Low-High = low-playing standard/high fitness; Low-Low = low-playing standard/low fitness. *a* denotes a moderate- to large-effect size difference between High-High and High-Low; *b* denotes a moderate- to large-effect size difference between High-Low and Low-Low; *c* denotes a moderate- to large-effect size difference between High-High and Low-High; and *d* denotes a moderate- to large-effect size difference between High-High and Low-Low.

Neuromuscular fatigue: There were reductions in CMJ power (ES = −0.75 to −2.37; *p* = 0.052) for each group over the competition (Figure 3A); these reductions were smallest in the two high-fitness groups from both playing standards (*p* = 0.340), with moderately greater reductions in CMJ power in the high-standard/low-fitness group compared with the high-standard/high-fitness group (ES = 0.74; likelihood = 85%, likely). In the high-standard group, there were greater reductions in CMJ power in the low-fitness players on day 1 (ES = 1.21; likelihood = 100%, almost certain) and day 3 (ES = 0.71; likelihood = 82%, likely). In the low-standard playing group, there was a similar, albeit less pronounced trend, with smaller reductions in CMJ power in the high-fitness group (ES = −0.41; likelihood = 66%, possibly).

Blood creatine kinase: There were increases in CK across the entire tournament for all groups (*p* = 0.001), although the smallest increases were seen in the high-standard/high-fitness group (Figure 3C). Compared with the high-standard/high-fitness group, there were moderately greater increases in blood CK in all other groups across the competition (high-standard/low-fitness group: ES = 0.63; likelihood = 76%, likely; *p* = 0.260; low-standard/high-fitness group: ES = 1.10; likelihood = 90%, likely; *p* = 0.623; low-standard/low-fitness group: ES = 0.95; likelihood = 87%, likely; *p* = 0.619). On days 2 and 3 of the competition, there was a greater increase in CK in the high-standard/low-fitness group (day 2, ES = 0.62; likelihood = 77%, likely; day 3, ES = 0.83; likelihood = 86%, likely), the low-standard/high-fitness group (day 2, ES = 0.93; likelihood = 90%, likely; day 3, ES = 1.00; likelihood = 92%, likely), and the low-standard/low-fitness group (day 3, ES = 1.14; likelihood = 94%, likely). On day 4 of the competition, there were greater increases in CK in both low-standard groups (high-fitness: ES = 0.95; likelihood = 94%, likely; low-fitness: ES = 0.97; likelihood = 92%, likely).

Perceptual wellbeing: There were reductions in perceptual wellbeing (*p* = 0.03) in each group over the course of the competition (Figure 3B). The greatest reductions were seen in the low-standard/high-fitness group, with similar reductions seen amongst the other groups. On day 1, there were smaller reductions in the high-standard/high-fitness group in comparison to all other groups. On day 2, there were smaller reductions in the low-standard/low-fitness group, with similar reductions amongst the other groups. On day 3, there was some recovery in perceptual wellbeing in all groups except the low-standard/high-fitness group. On day 4, there was little change in the two high-standard playing groups, but there were larger reductions in the low-standard playing groups.

## Discussion

This study compared the fatigue responses to intensified junior rugby league competition between playing standards. In addition, the influence that physical fitness had on the fatigue response and match activity profiles was also investigated. Our hypotheses were partially confirmed, with greater playing intensity in the high-standard playing group but little difference in fatigue responses between playing standards, although there was greater increases in blood CK in the low-standard playing group. Our second hypothesis was also partially confirmed with smaller increases in fatigue and blood CK as physical fitness increased despite greater playing intensities. As playing standard increases, so too do the physical demands of competition [15], although this does not appear to translate to increased player fatigue. Higher physical fitness is associated with greater playing intensity during an intensified competition and reduced post-match fatigue and markers of muscle damage.

The high-standard playing group had greater physical fitness than the low-standard group, highlighted by these players covering more metres on the Yo-Yo IRT. This is in accordance with previous research that has shown that as playing standard increases, so too do physical qualities [17,18]. Elevated physical fitness appeared to translate to increases in absolute and relative match workloads. High-standard players covered more metres per minute of match play, which was primarily achieved through greater moderate-speed running. Despite these greater match intensities in the high-standard playing group, when match speed was expressed relative to maximal speed on the Yo-Yo IRT, there was no difference in playing intensity between groups. This suggests that the match speeds in both the high- and low-standard competitions would have placed a similar relative physiological stress on players. On the other hand, the greater internal loads experienced by the high-standard players (highlighted by RPE and session RPE) are suggestive of greater physiological strain during the competition. This could be explained by the increased number of physical collisions and RHIE bouts performed by the high-standard group, which are overlooked by simply assessing match running intensities. Both collisions and RHIE bouts are extremely demanding tasks, both physically and mentally [33,34], and therefore could explain the increased internal loads in the high-standard playing group. These results clearly indicate that as playing standard increases, so too does physical fitness which appears to translate to increases in playing intensity [15,35]. In addition, only assessing the running demands of the game clearly underestimates the physiological loads placed on players.

When players were divided into high-fitness and low-fitness groups across both playing standards based on Yo-Yo IRT performance, there were some differences in absolute and relative playing intensities. In the high-standard group, there was a larger disparity between the high- and low-fitness groups in terms of match workloads. High-fitness players had greater playing minutes, covering more absolute and relative distance, primarily achieved through increased moderate-speed running. The match speeds performed in the high-fitness group were at a greater intensity relative to the maximal Yo-Yo speed. In addition, these players were involved in more collisions and RHIE bouts over the tournament. The differences in the low-standard playing group were less pronounced, with high-fitness players covering more metres per minute, primarily achieved through increased low-speed activity. The large differences in physical performance observed in the high-standard group and smaller differences observed in the low-standard group may be explained by the Yo-Yo IRT scores for each group. In the high-standard players, there was a larger absolute difference in Yo-Yo IRT scores between the high- and low-fitness groups than in the low-standard players. As such, maximising absolute high-intensity running ability, and introducing minimum standards, would be useful in order to maintain performances across the entire playing group.

Despite the increased absolute external and internal loads in the high-standard playing group, there were similar increases in fatigue and markers of muscle damage across the competition between playing standards. These increases in fatigue and markers of muscle damage were greatest over the first 2 days of the competition in both groups when players were involved in two games on each day. This suggests that physical fitness may offer some protective effect against fatigue and markers of muscle damage, which is in accordance with previous research [9]. Although there were greater reductions in CMJ power in the high-standard group, these differences were small in magnitude and did not reach the threshold of practical importance [30]. The similar changes in lower body fatigue between the high- and low-standard teams may be indicative of the similar match speeds relative to maximal Yo-Yo IRT. However, there were smaller increases in blood CK in the high-standard playing group, particularly at the end of days 2 and 4 of the competition. This result is even more significant given that the high-standard playing group was involved in 40% more collisions over the tournament, which are directly linked to elevations in CK [22]. There were also similar reductions in perceptual wellbeing over the competition between the playing standards. Despite greater absolute workloads in the high-standard playing group, there was little difference in the fatigue responses and smaller increases in blood CK across the tournament. This may be due to increased physical fitness in the high-standard playing group. In addition, expressing match speeds in relation to maximal Yo-Yo IRT speed may be a useful metric for determining the relative stress placed on each individual player by the running component of match play.

The protective effects of physical fitness on muscle function and to some extent muscle damage are clear when players were divided into low- and high-fitness groups. Despite greater match speeds in relation to maximal Yo-Yo IRT speed as well as absolute workloads, the high-fitness players in both playing standards exhibited the smallest reductions in CMJ power across the tournament. Previous research has suggested that players with well-developed high-intensity running ability possess greater eccentric strength and the ability to utilise the stretch-shortening cycle [36] which may result in less muscle fatigue [9]. High fitness only appeared to reduce muscle damage in the high-standard group. This group had significantly greater Yo-Yo IRT performance than the low-standard/high-fitness players (1,700 ± 119 m vs. 1,089 ± 188 m) which may suggest that, irrespective of playing intensity, there is a base level of fitness required before muscle damage is reduced through increased fitness. Once again, these high-fitness players performed more collisions than their less fit counterparts, clearly highlighting the protective effect of well-developed physical fitness to elevations in blood CK [9]. There was little difference in perceptual wellbeing between groups, although the low-standard/high-fitness group had greater reductions across the competition. Taken together, well-developed physical fitness appears to reduce post-match fatigue and increase match-playing intensities. As such, coaching staff should aim to maximise fitness prior to intensified competition in order to minimise post-match fatigue.

Whilst this study adds to the body of literature on fatigue in team sport players, our findings are not without their limitations. Only Yo-Yo IRT performance was assessed as a measure of physical fitness. Previous research indicates that lower body strength also impacts on post-match fatigue [9]. Therefore, it is likely that other physical qualities, such as muscular strength, may influence the fatigue response seen following intensified competition. In addition, data collection ceased immediately following the competition. As such, it is unknown whether the recovery time-course differed between groups. Future research addressing these points is warranted.

## Conclusions

This study highlighted that as playing standard increases in junior rugby league players, so too does the intensity of match play. Well-developed high-intensity running ability appears to be associated with greater absolute and relative internal and external workloads during competition. Despite increased workloads in players with well-developed physical qualities, these players experienced smaller increases in blood CK and less pronounced reductions in muscle function. As such, increasing fitness in junior players may be one of the most effective strategies for minimising accumulations in post-match fatigue and markers of muscle damage during intensified rugby league competition. Intensified competitions are always likely to occur in junior rugby league and numerous other team sports. Therefore, players should be exposed to demanding training prior to participating in tournaments in an attempt to increase physical fitness and gain a degree of protection against post-match fatigue.
